# Medication management of febrile children: a qualitative study on pharmacy employees’ experiences

**DOI:** 10.1007/s11096-016-0353-y

**Published:** 2016-07-23

**Authors:** Jacqueline P. G. Stakenborg, Eefje G. P. M. de Bont, Kirsten K. B. Peetoom, Marjorie H. J. M. G. Nelissen-Vrancken, Jochen W. L. Cals

**Affiliations:** 1 Department of Family Medicine, CAPHRI School for Public Health and Primary Care, Maastricht University, PO Box 616, 6200 MD Maastricht, The Netherlands; 2 Dutch Institute for Rational Use of Medicine, P.O. Box 3089, 3502 GB Utrecht, The Netherlands

**Keywords:** Anti-bacterial agents, Child, Community pharmacy, Fever, Netherlands, Prescription

## Abstract

*Background* While fever is mostly self-limiting, antibiotic prescription rates for febrile children are high. Although every parent who receives a prescription visits a pharmacy, we have limited insight into pharmacy employees’ experiences with these parents. Pharmacy employees do however exert an important role in ensuring children receive correct dosages and in advising parents on administration of antibiotics. *Objective* To describe pharmacists’ and pharmacy assistants’ experiences with parents contacting a pharmacy for their febrile child, and to identify ways of improving medication management of these children. *Setting* Community pharmacies in the Netherlands. *Method* A qualitative study including 24 Dutch pharmacy employees was conducted, performing four focus group discussions among pharmacy employees. Analysis was based on constant comparative technique using open and axial coding. *Main outcome measure* Pharmacy employees’ experiences with parents contacting a pharmacy for their febrile child. *Results* Three categories were identified: (1) workload and general experience, (2) inconsistent information on antibiotic prescriptions, (3) improving communication and collaboration. Pharmacy employees experienced that dosing errors in antibiotic prescriptions occur frequently and doctors provide inconsistent information on prescriptions. Consequently, they have to contact doctors, resulting in a higher workload for both stakeholders. They believe this can be improved by providing the indication for antibiotics on prescriptions, especially when deviating from standard dosages. *Conclusion* Pharmacy employees experience a high amount of dosing errors in paediatric antibiotic prescriptions. Providing the indication for antibiotics in febrile children on prescriptions, especially when deviating from standard dosages, can potentially reduce dosage errors and miscommunication between doctors and pharmacy employees.

## Impact of findings on practice

- Pharmacy employees believe that when GPs and other doctors provide the indication for antibiotics on the prescription, this can help reduce dosage errors and increase safety in the paediatric population.
- Mentioning the reason(s) for deviating from guidelines on choice and dosage of antibiotics might increase safety of paediatric medication.

## Introduction

Fever is a common symptom in children and the most common reason for parents to consult primary care services, especially during out-of-hours care [1, 2]. Guidelines are conservative concerning the use of antibiotics even in cases of fever with a focus, since fever is mostly self-limiting [3, 4]. Furthermore, parents generally do not expect an antibiotic prescription when consulting with their febrile child [5, 6]. Nevertheless, antibiotic prescription rates for febrile children in general practice are high, especially during out-of-hours care where one in three to four children receive an antibiotic [4, 7]. Re-consultations with a general practitioner (GP) during the same illness period are common and are associated with parental uncertainty and fear of complications. Parents experience a lack of knowledge on self-management strategies. Furthermore, a lack of consistency in the information given to patients may result in confusing advice [2, 8–10].

Previous studies showed that dosage errors in paediatric prescriptions are common. Children are exposed to a higher rate of dangerous medication errors compared to adults [11]. Furthermore, problems with administration of antibiotics occur in more than 30 %. Parents find it difficult to administer medication to their child and children tend to be more sensitive to side effects. Parents find it hard to continue prescribed medication when these side effects occur [12–14].

Dosing of antibiotics in children is complex for doctors [15]. In the 1940s, dosing was based on weight, from the 1960s also on age. These same dosing regimens seem to have been followed for the last 50 years. Currently there is a lack of recent evidence to support these recommendations, especially, since children’s body compositions have changed in the last decades, leading to many children being under-dosed. [15, 16]. Furthermore, the quality of prescribing varies amongst GPs [17]. High prescription rates, problems with antibiotic administration and incorrect dosing drive antimicrobial resistance, non-compliance, and ineffective treatment of febrile children [12].

Because dosing of antibiotics in children is complex, the pharmacy exerts an important role in medication management for children. They also play a central role in advising parents on correct antibiotics administration and how to deal with side effects. However, evidence with regards to what happens at the pharmacy following a GPs’ consultation is lacking. In order to improve medication management and antibiotic prescribing for febrile children, it is important to learn about pharmacy employees’ experiences with these children.

## Aim of the study

This qualitative study aims to study pharmacy employees’ experiences with parents contacting the pharmacy for a febrile child and to identify ways of improving medication management for these children.

## Ethics approval

This study was approved by the Medical Ethics Committee of the Maastricht University Medical Centre (NL METC 15-4-061). Participants’ data were encoded by numbering, ensuring anonymity of the included subjects. Written informed consent was obtained from all participants.

## Method

We performed a qualitative study based on naturalistic inquiry using focus group discussions with pharmacy employees to study their experiences with parents of febrile children contacting pharmacies [18].

### Setting

This study was carried out among pharmacists and pharmacy assistants: pharmacy employees from four different pharmacies in Limburg, the Netherlands. Focus group discussions were held at the participating pharmacies.

### Subjects

Pharmacists in the area were approached by email with the request to participate in this study. Focus groups were organized with a minimum of five subjects, including at least one pharmacist in each group. Employees from one pharmacy represented one group. We recruited pharmacies using purposeful sampling with the aim of achieving maximum variation between groups with regards to size of the pharmacy (client number), the number of pharmacy employees, and the community deprivation level. To obtain a more heterogeneous representation we included an out-of-hours pharmacy and pharmacies that had employees who previously worked out-of-hours. Out-of-hours pharmacies open only during the evening, nights and weekends.

### Data collection

Focus group discussions were used to generate insight into the experiences among pharmacy employees [19]. We prepared a topic list using sensitizing concepts. Questions were distilled into this topic list after literature research and a priori expert discussions [20]. Questions covered multiple aspects related to contacts with parents of febrile children at the pharmacy and medication management for these children. Covered topics were: workload and general experience, information provision, reasons for parents to contact the pharmacy, frequently asked questions/problems and medication management (prescriptions, dosing control). Data saturation was achieved after the third focus group. To validate the presumed saturation we performed one extra focus group. The discussions lasted 45–60 min and were facilitated by an independent moderator. Group dynamics and non-verbal communication were studied by two observers and noted in a research-diary. The discussions were audio-recorded and transcribed verbatim by JS.

### Data analysis

We analysed data using the constant comparison technique. Data collection and analysis took place simultaneously from February to April 2015 [20, 21]. Every focus group was analysed independently by two researchers (EB and JS), both present at the focus groups. Analysis was performed prior to the next focus group, thereby allowing room for refinement and adjustment of data collection. The topic list was discussed and adjusted several times among the wider research team [21]. Categories were derived using inductive content analysis, first using open and finally axial coding [20, 21]. NVivo software version 9.0 was used to facilitate data analysis. Discussion in the wider research team resolved inconsistencies by consensus.

### Trustworthiness

To enhance trustworthiness we embedded several strategies in our study. Data triangulation was used by including pharmacies with different sizes, areas and working hours. Methodological triangulation was enhanced by using a research-diary. The moderator had a different background (pharmacist) than the two researchers (medicine), strengthening the investigator triangulation. Data collection and analysis were performed by two researchers independently. Peer debriefing was organized with the wider research team. A member check of the written transcript was performed among the participants. In order to let others decide to what extent the results of this study are transferable to their context, we provided a detailed description of the methodology and subjects included. An audit trail was created to allow for replicability [22]. We used the criteria included in Consolidated criteria for reporting qualitative research (COREQ) to report important aspects of the research team, study methods, context of the study, findings, analysis and interpretations (Table 2, Appendix).

## Results

Nine pharmacies were approached; six consented to participate of which four were used for a focus group before saturation was reached. A lack of time was given as the reason for those not consenting to participate. We included three regular pharmacies, one of which also has opening hours on Saturdays, and one out-of-hours pharmacy. Pharmacies from rural and urban areas were included and varied in size with respect to the number of employees and clients. Five pharmacists and 19 pharmacy assistants participated (2 male, 22 female). Mean age was 39 years (range 23–64 years), average years of working experience was 17 (range 0–42 years), 7 of the 19 pharmacy employees working at the regular pharmacies (37 %) also had experience of working out-of-hours.

**Fig. 1:**
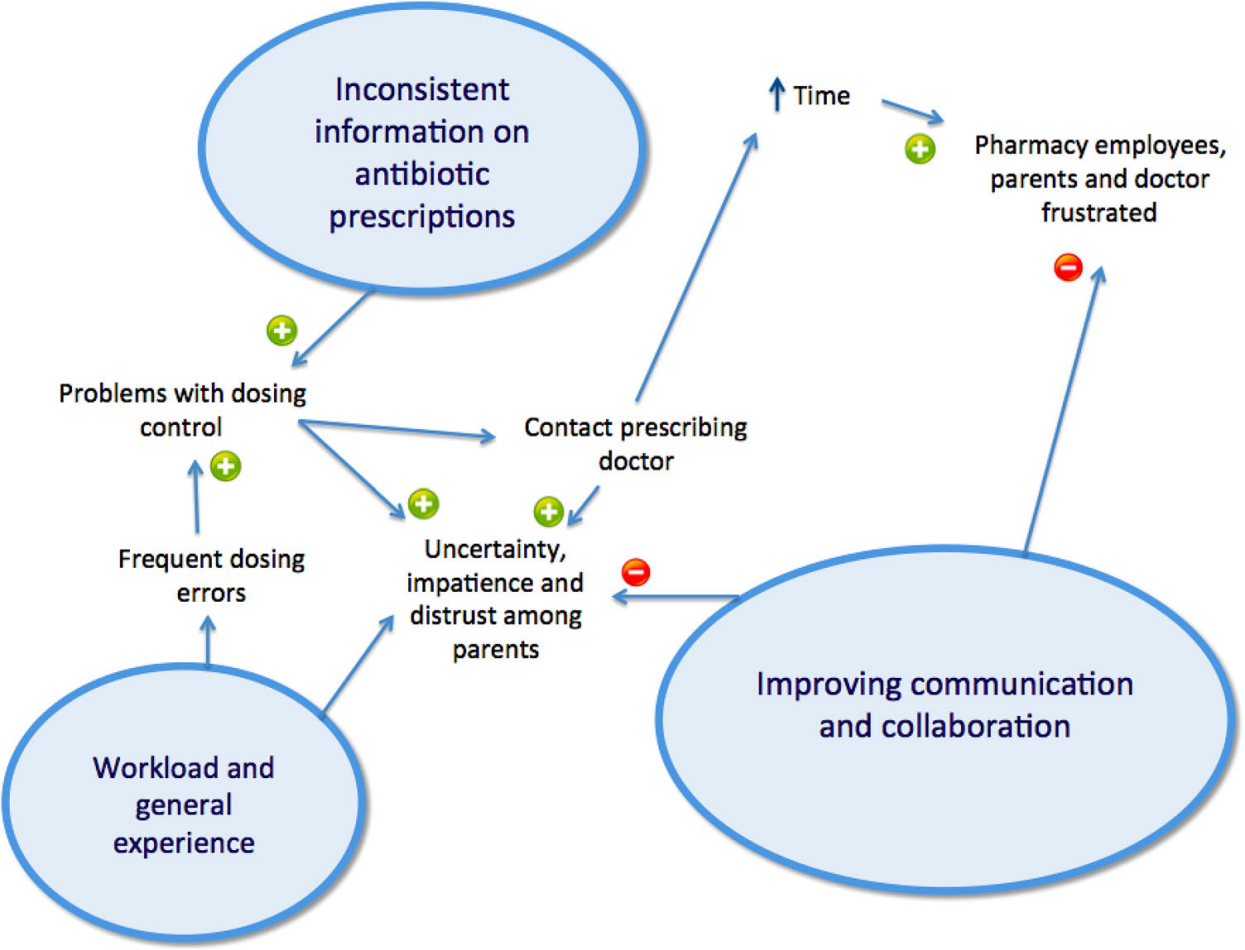
Identified categories—all closely interwoven: workload and general experience, inconsistent information on antibiotic prescriptions and improving communication and collaboration

We identified three main categories from the data: (1) workload and general experience, (2) inconsistent information on antibiotic prescriptions, (3) improving communication and collaboration. Figure 1 shows an overview of the main categories. Table 1 shows a tabulated form of the identified categories and the respondents’ quotes.

**Table 1:** Tabulated form of the identified categories and the respondents’ quotes

Identified category	Respondents’ quotes
*Workload and general experience*	
Workload	*“*Coincidentally, I checked it [the number of antibiotic prescriptions for children] last weekend. I stopped counting when I got to 26 amoxicillin prescriptions starting from Friday night until Sunday morning. After this, there were at least another 5-6 prescriptions, so in total around 30 amoxicillin prescriptions for children*.”* (FG 2, pharmacy employee (PE) 3, pharmacy assistant)
Workload	*“*We do see a lot of parents of febrile children, especially in the winter period, when the rate of infections is higher*.”* (FG 2, PE 11, pharmacy assistant)
General experience	“Yes and they want to go home with their child because they were waiting in the doctor’s waiting room, and then you still have to prepare it [the prescription] and they have to wait for this. So I constantly feel the impatience of these parents when I am doing this.” (FG 3, PE 13, pharmacist)
General experience	“And we would like to explain something. Like today, the doctor wrote 2 millilitres, 3 times a day, a prescription for a completely different dosage to the one we will deliver. So they [the parents] will have to administer 4 millilitres, 3 times a day, so you want to explain this carefully. Parents will not ask anything, they just want to go home and they think: ‘Yes I know everything.’ But then, a few days later they contact us, stating that the dosage we provided was incorrect.” (FG 3, PE 13, pharmacist)
General experience	“I feel parents are sometimes distrustful towards us: ‘Yes, but didn’t the doctor write that down?!’” (FG 2, PE 11, pharmacy assistant)
*Inconsistent information on antibiotic prescriptions*	
Inconsistency in providing prescriptions, incomplete prescriptions	“Most of the time, they just write down: ‘10 kilograms, please calculate’.” (FG 1, PE 3, pharmacy assistant)
Dosage errors	“There is almost no doctor’s prescription that is correct anymore.” (FG 1, PE 5, pharmacy assistant)
	“Do you feel limited by not knowing certain information?” (moderator) “Absolutely.” (FG 1, PE 1, pharmacy assistant)
	“This [having a discussion about a dosage with a doctor] also makes parents insecure.” (FG 3, PE 18, pharmacy assistant)
	“Yes, and what are you supposed to do then, should you under-dose? No, then you unfortunately have to contact them again and hope they won’t be angry. And ask if it [the dosage] could please be a little bit higher.” (FG 1, PE 5, pharmacy assistant)
Improving communication and collaboration	“Because you often don’t know the reason why a doctor advises a particular dosage, so indeed, you have to contact them.” (FG 2, PE 11, pharmacy assistant)
	“It would be a lot more convenient if they provided the indication on the prescription. In this way we would be able to organize it much easier.” (FG 3, PE 17, pharmacy assistant)
	“When you have contacted a specialist doctor, put this in the free text. It just takes a small effort and it saves us both the effort of having a phone call.” (FG 3, PE 13, pharmacist)

### Workload and general experience

Pharmacy employees working during office hours experienced a minimal workload imposed by parents contacting them for their febrile child. In contrast, pharmacy employees working out-of-hours perceived a strikingly higher workload and stated that antibiotic prescriptions for febrile children, mostly prescribed by GPs, are one of the most frequent prescribed medications. Pharmacy employees with experience of both types of services confirmed an evident difference in workload between them.

Coincidentally, I checked it [the number of antibiotic prescriptions for children] last weekend. I stopped counting when I got to 26 amoxicillin prescriptions starting from Friday night until Sunday morning. After this, there were at least another 5-6 prescriptions, so in total around 30 amoxicillin prescriptions for children. (FG 2, pharmacy employee (PE) 3, pharmacy assistant)

They stated that they observe a seasonal influence and difference between age categories.

We do see a lot of parents of febrile children, especially in the winter period, when the rate of infections is higher. (FG 2, PE 11, pharmacy assistant)

They explained that parents of febrile children contact the pharmacy either with an antibiotic prescription or for over-the-counter (OTC) drugs, rarely for advice. They perceived parents don’t contact a pharmacy but rather a GP when problems occur with administration of medication or when their child has side effects from antibiotics.

They experienced that once parents contact the pharmacy, they seem impatient and restless, especially during out-of-hours care. According to them, this might be caused by the fact that they have been waiting at the doctor’s office and then at the pharmacy so want to go home with their child as soon as possible.

Yes and they want to go home with their child because they were waiting in the doctor’s waiting room, and then you still have to prepare it [the prescription] and they have to wait for this. So I constantly feel the impatience of these parents when I am doing this. (FG 3, PE 13, pharmacist)

Pharmacy employees expressed understanding for this impatience and restlessness but also felt that this might add to suboptimal information provision for these parents about the prescribed medication and/or care for their child. They explained they have the perception that during a GP’s consultation little attention is paid to the fact that pharmacies are important to inform parents about this.

And we would like to explain something. Like today, the doctor wrote 2 millilitres, 3 times a day, a prescription for a completely different dosage to the one we will deliver. So they [the parents] will have to administer 4 millilitres, 3 times a day, so you want to explain this carefully. Parents will not ask anything, they just want to go home and they think: ‘Yes I know everything.’ But then, a few days later they contact us, stating that the dosage we provided was incorrect. (FG 3, PE 13, pharmacist)

Some perceived that this also contributes to their feeling that parents are distrustful towards them and sometimes irritated when dosages are checked and/or adjusted, questions are asked and when different and/or additional information is provided with regards to what the doctor explained.

Pharmacy employees experienced that parents in general attach more credibility to what the doctor has told them compared to what they are trying to explain. This makes it difficult for them to give advice and adjust medication management, while this is often necessary and one of their primary tasks. Pharmacy employees expressed their frustration.

I feel parents are sometimes distrustful towards us: ‘Yes, but didn’t the doctor write that down?!’ (FG 2, PE 11, pharmacy assistant)

### Inconsistent information on antibiotic prescriptions

Pharmacy employees experienced that prescribing doctors are inconsistent and often incomplete with regards to what information they provide on antibiotic prescriptions for children.

Most of the time, they just write down: ‘10 kilograms, please calculate’. (FG 1, PE 3, pharmacy assistant)

There is inconsistency with regards to whether doctors calculate the dosage and whether they mention the indication for the antibiotic on the prescription. Furthermore, pharmacy employees stated that dosage errors, as in errors in the calculated dosage provided on the prescription by the GP according to guidelines, occur frequently in paediatric antibiotic prescriptions.

There is almost no doctor’s prescription that is correct anymore. (FG 1, PE 5, pharmacy assistant)

Frequent dosage errors and inconsistent information on antibiotic prescriptions result in problems when checking them. They perceived no problems with the correction of the dosage itself since all pharmacies follow the same guidelines but problems do arise from the fact that they often lack relevant information on prescriptions for revision of the dosage.

Do you feel limited by not knowing certain information? (moderator)

Absolutely. (FG 1, PE 1, pharmacy assistant)

Consequently, pharmacy employees frequently have to consult the prescribing doctor, resulting in a higher workload for both stakeholders. They explained this leads to frustration and/or irritation for pharmacy employees and most likely for prescribing doctors, and parents. Furthermore they experienced that parents seem to find it confusing when there is discussion about a prescription after a doctor’s visit and that this leads to parental uncertainty.

This [having a discussion about a dosage with a doctor] also makes parents insecure. (FG 3, PE 18, pharmacy assistant)

In some pharmacies, employees expressed tension regarding these fever-related contacts and experienced this as a burden. They perceived that some doctors feel criticized or irritated when they consult them about a dosage or indication for an antibiotic.

Yes, and what are you supposed to do then, should you under-dose? No, then you unfortunately have to contact them again and hope they won’t be angry. And ask if it [the dosage] could please be a little bit higher. (FG 1, PE 5, pharmacy assistant)

### Improving communication and collaboration

As was mentioned, prescribing doctors are contacted when there are questions about the prescribed antibiotic and/or the amount of the dosage.

Because you often don’t know the reason why a doctor advises a particular dosage, so indeed, you have to contact them. (FG 2, PE 11, pharmacy assistant)

Pharmacy employees stated that it would be timesaving and beneficial if doctors mentioned the indication for the antibiotic, especially when deviating from standard dosages. It would facilitate double-checking dosages and prescriptions, thereby increasing medication safety for these children and reducing unnecessary contact with prescribing doctors. Since most prescriptions are provided by GPs, they believed this message would be most relevant for them.

It would be a lot more convenient if they provided the indication on the prescription. In this way we would be able to organize it much easier. (FG 3, PE 17, pharmacy assistant)

They also explained that sometimes when contacting the prescribing GP, it appears that the doctor deviated from the standard dosage after consultation with a specialist doctor. In these cases, pharmacy employees found it even more important to mention this on a prescription, thereby avoiding miscommunication.

When you have contacted a specialist doctor, put this in the free text. It just takes a small effort and it saves us both the effort of having a phone call. (FG 3, PE 13, pharmacist)

In some pharmacies there were already specific agreements between the pharmacy and the doctors. These agreements allowed pharmacy employees to correct the antibiotic dose in cases of under-dosing and in some pharmacies mentioning the indication for the antibiotic on the prescription was already incorporated in their work process.

## Discussion

### Summary of the main results

Pharmacy employees report that they see a lot of parents with antibiotic prescriptions for their febrile child during out-of-hours care, mostly provided by GPs. Errors in dosing are strikingly common in paediatric antibiotic prescriptions and doctors are inconsistent with regards to the information they provide on antibiotic prescriptions. This can decrease the pharmacist’s ability to check the dosage on a prescription, leading to a risk of unsafe medication management in these children and frequent contacts with prescribing doctors which likely leads to frustration for all those involved. Pharmacy employees suggest that if we want to improve medication management for febrile children, doctors and especially GPs should consider providing an indication on prescriptions, especially when deviating from standard dosages.

### Strengths and limitations

This is the first qualitative study that provides an in-depth insight into pharmacy employees’ experiences with parents of febrile children. The results of this study give clear guidance for the improvement of medication management for febrile children.

Despite efforts to make participants feel comfortable and safe by conducting the focus group discussions in their work environment, they may have given socially acceptable answers, thereby holding back valuable information. The different perspectives, member check, peer debriefings, investigator and data triangulation did, however, help us to increase trustworthiness.

Since health care systems are culturally different, we do not know to what extent these results are transferable to other countries. They are likely not transferable to countries where antibiotics can be bought over-the-counter. Also, in some other countries is it already required to mention the indication on prescriptions. However, we did use purposeful and heterogenic sampling and the path from GP’s office to pharmacy is common in other countries. We provided a detailed explanation of our methods and sample, allowing others to decide on transferability to their contexts [23].

### Comparison with existing literature

Previous research has shown that the attendance rate of febrile children at primary care services is high, especially out-of-hours [1, 2]. This study shows that pharmacy employees experience the same. An explanation for this might be that antibiotic prescription rates for febrile children are higher during out-of-hours care compared to the rate during office hours [4, 24].

Previous studies aimed at improving safety in antibiotic medication management in children were mainly performed in secondary, paediatric care settings, where medication management is much more controlled than in a primary care setting [17, 25].

Although the recommendations from this study might be partially applicable to other patient groups, they are specifically formulated for febrile children in primary care. Since prescriptions and consultations are high, but more importantly because dosing is complex and dosing errors occur frequently in this group [11, 26].

Mentioning the indication on prescriptions might reduce patients’ privacy. To our opinion, more efficient collaboration between pharmacy employees and doctors does however counterbalance this since safety for febrile children might be enhanced. Requiring indications being written on all prescriptions is already implemented in health care systems of other countries than the Netherlands. In the Netherlands this is only required for certain medications, not yet for antibiotics. Mentioning the indication on prescriptions is also known to have a positive impact on patient safety [27]. This study shows that collaboration between GPs and pharmacies is not only crucial in the management of a chronic disease but for all patient groups [17, 28].

### Implications for research and practice

Pharmacy employees perceived that parents visiting a pharmacy are restless, impatient and distrustful towards them. This was not earlier described in literature. Future research should further investigate parental experiences with pharmacies. It should also focus on implementing a standardized system with regards to information provided on antibiotic prescriptions for children by GPs. Future research must focus on how information provision at pharmacies might be improved.

The following concrete ideas for improvement of prescriptions were proposed: (1)mentioning the indication for the antibiotic prescription at least when deviating from standard dosages, (2)mentioning any prior consultation with a specialist doctor about the dosage or other reasons for deviating from guidelines on choice and dosage of antibiotics.

## Conclusion

Pharmacy employees experience frequent dosing errors in paediatric antibiotic prescriptions and feel doctors are inconsistent with regards to the information they provide on prescriptions. According to them, providing an indication for an antibiotic prescription in febrile children, especially when deviating from standard dosages, can potentially increase safety in medication management for febrile children by reducing dosage errors and miscommunication between doctors and pharmacies.

## Appendix

See Table 2.

**Table 2:** Consolidated criteria for reporting qualitative studies (COREQ): 32-item checklist

**Domain 1: Research team and reflexivity**	
*Personal characteristics*	
1. Interviewer/facilitator	Which author/s conducted the interview or focus group? Jacqueline P.G. Stakenborg, Eefje G.P.M. de Bont, Marjorie H.J.M.G. Nelissen-Vrancken
2. Credentials	What were the researcher’s credentials? E.g. PhD, MD Jacqueline P.G. Stakenborg, MD, MSc Eefje G.P.M. de Bont, MD, MSc Marjorie H.J.M.G. Nelissen-Vrancken, PhD
3. Occupation	What was their occupation at the time of the study? Jacqueline P.G. Stakenborg: Medical trainee. Eefje G.P.M. de Bont: GP trainee and PhD student Kirsten K.B. Peetoom: PhD student Marjorie H.J.M.G. Nelissen-Vrancken: senior pharmacist and project leader with the National Institute for Rational Use of Medicine Jochen W.L. Cals: GP, PhD and researcher
4. Gender	Was the researcher male or female? Males and females
5. Experience and training	What experience or training did the researcher have? Jacqueline P.G. Stakenborg: Bachelor of Science Molecular Life Science; Physician-clinical investigator master track (MD, MSc) Eefje G.P.M. de Bont: PHD. Physician-clinical investigator master track (MD, MSc) Marjorie H.J.M.G. Nelissen-Vrancken: PhD, pharmacist
*Relationship with participants*	
6. Relationship established	Was a relationship established prior to study commencement? Only based on telephone and mail contact with the question whether they would like to participate in our study
7. Participant knowledge of the interviewer	What did the participants know about the researcher? e.g. personal goals, reasons for doing the research The reasons for doing the research
8. Interviewer characteristics	What characteristics were reported about the interviewer/facilitator? e.g. Bias, assumptions, reasons and interests in the research topic The moderator is a pharmacist. The two observers are MDs. All are of course interested in this subject. Bias possibly created by either the doctors or the pharmacist is made even by means of discussion afterwards in our research group
**Domain 2: study design**	
*Theoretical framework*	
9. Methodological orientation and theory	What methodological orientation was stated to underpin the study? e.g. grounded theory, iscourse analysis, ethnography, phenomenology, content analysis Principles of grounded theory. Inductive content analysis
*Participant selection*	
10. Sampling	How were participants selected? e.g. purposive, convenience, consecutive, snowball By means of purposeful and heterogenic sampling
11. Method of approach	How were participants approached? e.g. face-to-face, telephone, mail, email Telephone, mail
12. Sample size	How many participants were in the study? 24 participants
13. Non-participation	How many people refused to participate or dropped out? Reasons? 3 pharmacies. Lack of time
*Setting*	
14. Setting of data collection	Where was the data collected? e.g. home, clinic, workplace At the pharmacy
15. Presence of non-participants	Was anyone else present besides the participants and researchers? No
16. Description of sample	What are the important characteristics of the sample? e.g. demographic data, date Pharmacies from rural and urban areas were included and varied in size with respect to the number of employees and clients. Five pharmacists and 19 pharmacy assistants participated (2 male, 22 female). Mean age was 39 years (range 23–64 years), average years of working experience was 17 (range 0–42 years), 7 of the 19 pharmacy employees working at the regular pharmacies (37 %) also had experience of working out-of-hours
*Data collection*	
17. Interview guide	Were questions, prompts, guides provided by the authors? Was it pilot tested? Yes it was provided by the authors. It was not pilot tested but it was discussed in our research team
18. Repeat interviews	Were repeat interviews carried out? If yes, how many? No
19. Audio/visual recording	Did the research use audio or visual recording to collect the data? Yes. Audio and visual recording
20. Field notes	Were field notes made during and/or after the interview or focus group? Yes
21. Duration	What was the duration of the interviews or focus group? Approximately 1 h
22. Data saturation	Was data saturation discussed? Yes
23. Transcripts returned	Were transcripts returned to participants for comment and/or correction? Yes
**Domain 3: analysis and findings**	
*Data analysis*	
24. Number of data coders	How many data coders coded the data? Two
25. Description of the coding tree	Did authors provide a description of the coding tree? Yes
26. Derivation of themes	Were themes identified in advance or derived from the data? In advance but also after the first focus groups
27. Software	What software, if applicable, was used to manage the data? NVivo software version 9.0
28. Participant checking	Did participants provide feedback on the findings? No
*Reporting*	
29. Quotations presented	Were participant quotations presented to illustrate the themes/findings? Was each quotation identified? e.g. participant number Yes, they were presented to illustrate the findings. We coded every quotation (participant, focus group)
30. Data and findings consistent	Was there consistency between the data presented and the findings? Yes
31. Clarity of major themes	Were major themes clearly presented in the findings? Yes
32. Clarity of minor themes	Is there a description of diverse cases or discussion of minor themes? Yes there is
